# Modulation of Depolymerizable Poly(thioether-thioester) Properties in Reversible Covalent Composites

**DOI:** 10.1021/acsmacrolett.5c00422

**Published:** 2025-09-19

**Authors:** Binoy Maiti, Mridula Nandi, Jaehyun Cho, Liang Yue, Kellie Stellmach, Blair Brettmann, Qi Jerry, Will Gutekunst, M. G. Finn

**Affiliations:** †School of Chemistry and Biochemistry, ‡School of Chemical and Biomolecular Engineering, §George W. Woodruff School of Mechanical Engineering, and ∥School of Materials Science and Engineering, 1372 Georgia Institute of Technology, Atlanta, Georgia 30332, United States

## Abstract

We incorporated thiol-functionalized silica particles as macroinitiators for the construction of composites by ring-opening polymerization of thiolactones. A separate photochemical cross-linking step was employed to enhance the stability of the polymer composite material. The thermal and mechanical properties of the materials can be tuned by varying the amount of particles, and a representative formulation could be 3D printed. The polymer composite was depolymerized in the presence of a catalytic amount of thiol and 1,8-diazabicyclo(5.4.0)­undec-7-ene (DBU) base to recover substantial amounts of monomer, which were repolymerized and photo-cross-linked to give a material very similar in mechanical properties to the virgin composite. The modular nature of this system and the reliability of the bond-forming and bond-breaking steps suggest that it may prove to be useful as a new type of recyclable plastic.

## Graphical abstract

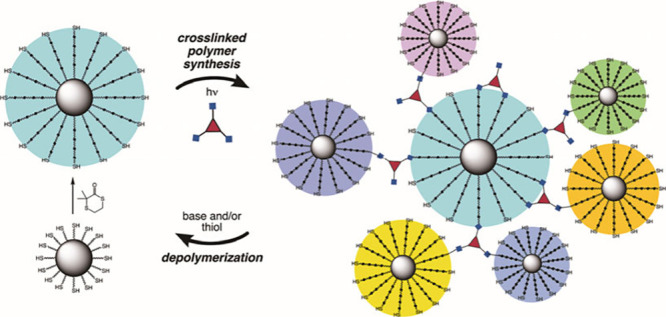

The recycling of bulk plastics is an important goal for sustainability, energy, and environmental concerns. However, the goals of mechanical and chemical stability seem at odds with the goal of recyclability, with the former requiring strong and lasting bonds, and the latter requiring their breakage. Industrial-scale mechanical recycling often suffers from a significant loss of polymer properties.^1,2^ An alternative approach is chemical recycling in which the polymer is depolymerized under conditions different from those in which the material is used, allowing the recovery of monomers that can be subsequently repolymerized to get virgin-quality polymeric materials.^3–7^

One approach to depolymerizable materials employs reversible bond formation in polymer synthesis, placing the integrity of the material in the hands of thermodynamics. The ceiling temperature (*T*_c_) of such materials is defined as the temperature at which polymerization and depolymerization occur at the same rate, depolymerization thereby being favored at higher temperatures. Common bulk polymers, such as polyolefins, are known for their high *T*_c_ values, making depolymerization either energy-intensive or prone to decomposition. Lower *T*_c_ materials are thereby better candidates for chemical recycling to a monomer. Numerous monomers have been examined for their applicability in the synthesis of low *T*_c_ polymers, including lactones,^8–13^ thiolactones,^14–16^ phthalaldehydes,^17^ carbonates,^18,19^ and cyclic acetals.^20^ When *T*_c_ exceeds the polymerization temperature, catalysts are often used to trigger depolymerization, even above the critical temperature. For example, poly­(γ-butyrolactone) (PγBL) requires heating at 300 °C for depolymerization in the absence of catalysts, whereas the introduction of an organic or metal catalyst allows depolymerization to occur rapidly, even at room temperature. For example, PγBL exhibited *T*_c_ = −9 °C at [γ-BL]_0_ = 10 M, and *T*_c_ = −136 °C at [γ-BL]_0_ = 1.0 M.^21^ Similarly, while poly­(trithiocarbonate)­s made by ring-opening polymerization (ROP) of seven-membered cyclic trithiocarbonates can be depolymerized at 250 °C without catalyst,^22^ the depolymerization of several dithiolactone-based polymers is accelerated by DBU and thiol at lower temperatures.^23^

Low *T*_c_ materials often lack sufficiently robust physical and mechanical properties for practical, common applications. Functional fillers and reinforcements are often used to enhance such properties in polymer composites while maintaining flexibility, including nanostructures such as metal nanoparticles, metal oxides, three-dimensional porous nanofillers, and a wide range of carbon materials, most notably including graphene^24–26^ and carbon nanotubes^27,28^ due to their high aspect ratios, mechanical stability, and impressive thermal and electrical conductivity. A popular inorganic nanofiller is silica (SiO_2_), with the advantages of adaptable morphology, expansive specific surface area, ease of functionalization, and cost-effectiveness.

The enhancement of the overall performance in polymer–silica nanocomposites is contingent upon the careful consideration of surface modification, structural characteristics, and bonding mechanisms of SiO_2_. In recent years, many such advanced nanocomposites have been documented,^29–32^ but recyclability remains a major challenge.^33^ We describe here the exploration of nanoparticulate silica combined with a low-*T*_c_ poly­(thiolactone) polymer. We compare materials made by ring-opening ROP of cyclic thiolactone using a thiol-functionalized silica particle (SiO_2_@SH) with a standard composite formulation in which nonfunctionalized silica is mixed with the bulk polymer. We also describe the UV cross-linking of these materials, which produces composites with good thermal and mechanical properties tuned by varying the amount of particle and cross-linker. 3D printing, depolymerization, and repolymerization were demonstrated with mechanical properties comparable to the virgin composite.

Thiol-modified SiO_2_ (SiO_2_@SH): Spherical silica nanoparticles (average diameter = 20 nm) were functionalized by a standard sol–gel procedure under an inert atmosphere using (3-mercaptopropyl)­trimethoxysilane (MPS) to obtain thiol-modified particles designated as SiO_2_@SH. X-ray photoelectron spectroscopy (XPS, Figure S1) showed new signals for carbon and silicon, as expected. ^29^Si cross-polarization magic angle spinning (CP/MAS) NMR data confirmed the attachment of additional silicon atoms to the surface of the T^2^ and T^3^ type (Figure S3), observed as new peaks, in addition to Q^2^, Q^3^, and Q^4^ Si centers observed for the basic silica (where the superscript indicates the number of siloxane Si–O bonds).^34,35^ Thermogravimetric analysis (TGA, Figure S2) showed a 6.0% weight loss in excess of that observed for bare silica at 700 °C, which translates^36,37^ to a value of approximately 0.58 mmol of thiol groups per gram of particle. This represents the monolayer occupancy of the theoretical number of surface SiOH groups, assuming that the surface area is as reported by the manufacturer (approximately 640 m^2^/g).

Synthesis and characterization of polymer composites: Consistent with the prior report of ring-opening polymerization of thiolactone **1** using thiol as initiator and DBU as a catalyst,^23^ we employed thiol-functionalized silica as a macroinitiator. The use of different amounts of silica (from 6 to 25 wt %) produced materials (**P1**, **P2**, and **P3**) of different bulk properties (gummy to rubbery), as shown in Figure 1. These were compared to the cyclic polymer^22^ made in the presence of DBU without initiator (**P0**), the standard linear polymer made with an organic thiol initiator (**P5**), and a mixture of **P0** with 12 wt % of unfunctionalized silica (designated **P4**).

**Figure 1:**
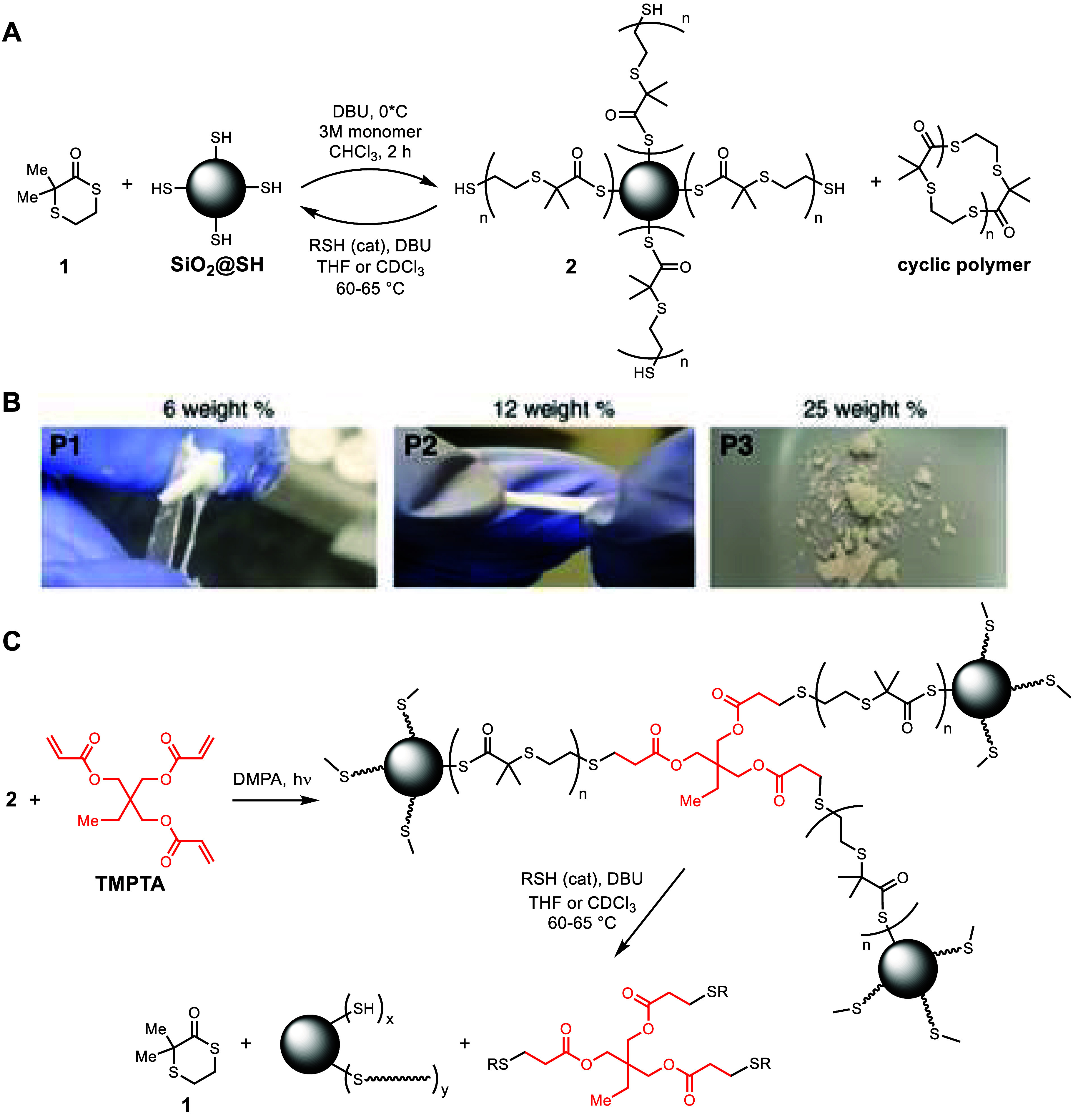
(A) Use of SiO_2_@SH as a macroinitiator in thiolactone ring opening polymerization and depolymerization. (B) Representative pictures of the materials made with the indicated amounts of SiO_2_@SH macroinitiator. **P1** has a wet, gel- or gum-like consistency, **P2** is a flexible, stretchable material, and **P3** is a brittle solid. (C) Photo-cross-linking of composite polymers and depolymerization of **P8**.

TGA and derivative thermogravimetric (DTG) characterization of these composite materials are shown in Figures S4 and S5 and summarized in Table 1. After loss of residual adsorbed water (<2%) below 200 °C, the onset of thermal decomposition was found to be 30–50 °C higher for the silica-containing materials than for the purely organic materials. The comparison of **P2** with P5* provides the best test of covalent vs noncovalent incorporation of the nanoparticles, as they both have the same particle content (∼12 wt %). However, the same values of *T*_onset_ (308 ± 9 °C for **P2** and 302.3 ± 1.3 °C for P5*), and *T*_D50_ values (314 ± 2.3 °C for **P2** and 311 ± 0.9 °C for P5*) were observed, within experimental error, and glass transition temperatures of all samples were quite similar, whether or not they contained silica particles. The similarity in *T*_g_ values of **P0** and **P1** suggests that **P1** may contain significant amounts of cyclic polymer due to the competitive rates of the initiator-free (DBU catalyzed) cyclopolymerization and macroinitiated linear polymerization.^38^

**Table 1:** Summary of the Thermal Properties of Different Polymer Composites from TGA and DSC Analyses

sample	composition	*T*_onset_ (°C)	*T*_D50_ (°C)	*T*_max_ (°C)	*T*_g_ (°C)
**P0** ^a^	**1**/no particle	251	289	303	–15.8
**P1**	**1**/SiO_2_@SH (6 wt %)	287	304	310	–15.3
**P2**	**1**/SiO_2_@SH (12 wt %)	303	314	329	–11.3
**P3**	**1**/SiO_2_@SH (25 wt %)	280	311	315	–11.1
**P4** ^b^	**P0**/SiO_2_ (12 wt %)	280	303	309	–15.9
**P5** ^c^	**1**/RSH	258	281	306	–14.0
P5*^d^	**P5**/SiO_2_ (12 wt %)	301	310	313	–13.5
**P6**	**P0**/TMPTA^e^	299	323	322	
**P8**	**P2**/TMPTA^e^	318	327	327	
**P9**	**P4**/TMPTA^e^	318	329	326	

a [**1**]/[DBU] = 100/1, no particle added. wt % within parentheses corresponds to that of SiO_2_ and SiO_2_@SH in the composite.

b **P4** is a physical mixture of **P0** and 12 wt % SiO_2_.

c [**1**]/[DBU]/RSH = 100:1:1, RSH = dodecanethiol, no particle added.

d P5* is a physical mixture of **P5** and 12 wt % SiO_2_.

e monomer:TMPTA molar ratio = 10:1.

Cross-linked composites: Taking advantage of the presence of free thiols at the polymer chain ends, we created cross-linked materials using trimethylolpropane triacrylate (TMPTA) as a cross-linker and 2,2-dimethoxy-2-phenylacetophenone (DMPA or Irgacure 819) as a photoinitiator (Figure 1C). This efficient reaction was completed within 30 s for a series of polymer compositions (**P6**–**P10**), as summarized in Table 2. (The **P8** formulation was characterized by FTIR before and after irradiation, showing the expected disappearance of the acrylate C=C bond, and by gel fraction (72%), indicating significant cross-linking.) The resulting materials exhibited two-stage thermal degradation profiles by TGA (Figure 2A,B), assigned to successive thioester depolymerization and cross-linker retro-Michael and/or decomposition reactions.

**Table 2:** Comparison of Mechanical and Tensile Properties of Different Polymer Composites

polymer	composition	tensile strength (MPa)	Young’s modulus	strain at break	stress at break
**P6** ^a^	**P0**/TMPTA	0.46 ± 0.10	2.90 ± 0.47	0.195	0.338
**P7** ^a^	**P1**/TMPTA	0.50 ± 0.11	5.43 ± 0.51	0.185	0.563
**P8** ^a^	**P2**/TMPTA	1.130 ± 0.01	14.57 ± 0.35	0.162	1.103
**P9** ^a^	**P4**/TMPTA	0.44 ± 0.02	10.18 ± 0.51	0.142	0.462
**P10** ^b^	**P2**/TMPTA	0.52 ± 0.07	2.32 ± 0.01	0.590	0.501

a [monomer repeating unit:TMPTA] = 10:1.

b [monomer repeating unit:TMPTA] = 10:0.8.

**Figure 2:**
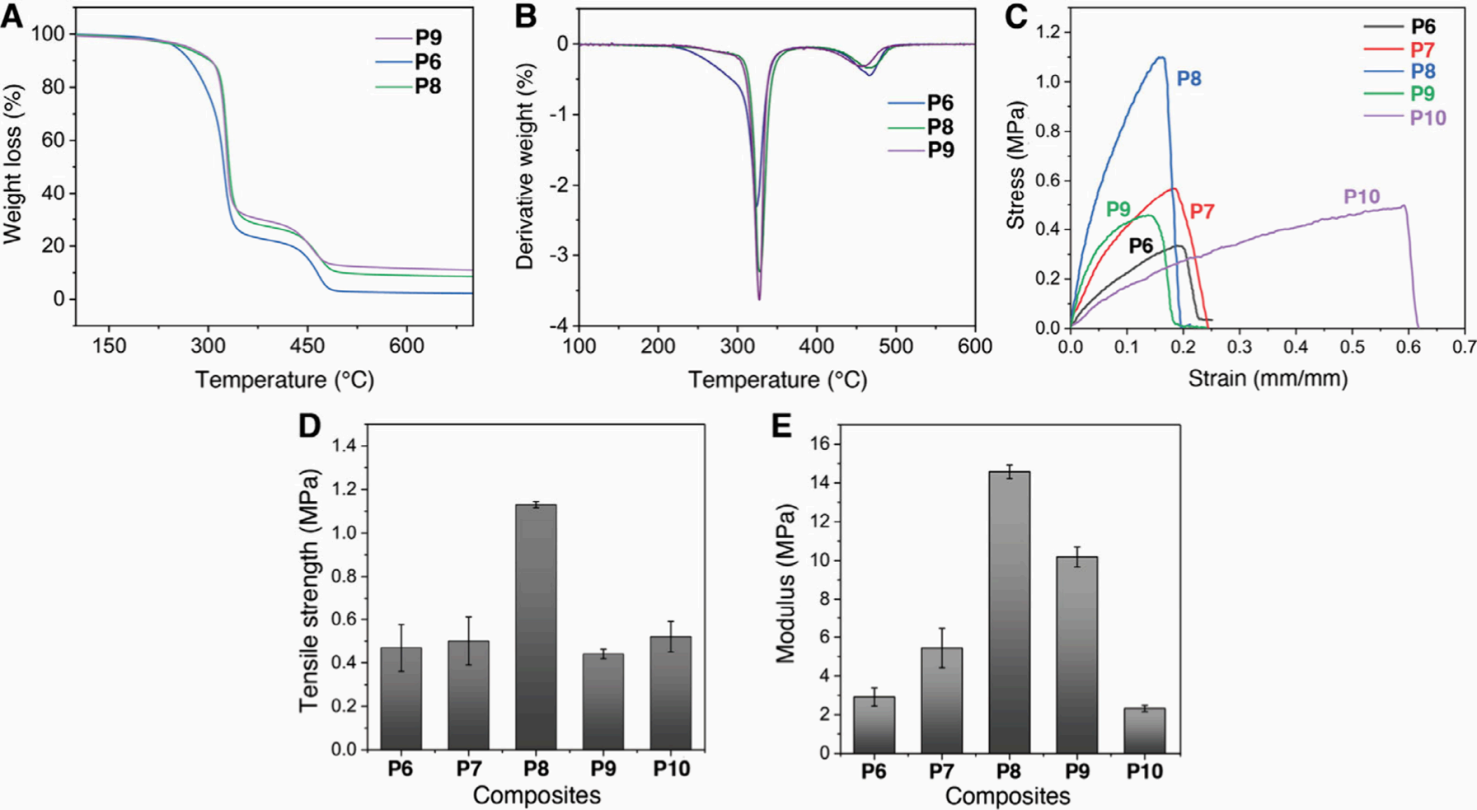
Thermochemical characterization of cross-linked composites: (A) TGA, (B) DTG thermograms, (C) stress–strain to break, (D) tensile strength, (E) Young’s modulus. Error bars represent standard deviation from three independent measurements.

Universal tensile testing of cross-linked materials **P6**–**P10** (Table 2) showed the cross-linked **P8** composite to display the highest tensile strength, modulus, and rigidity. That **P8** (12 wt % SiO_2_@SH) was significantly stronger than both **P7** (6 wt % SiO_2_@SH) and **P9** (12 wt % SiO_2_) suggests an active role of covalent cross-linking of the nanoparticle into the polymer matrix in modulating bulk properties. This is further highlighted by the significant difference in bulk properties between **P8** and **P10**, which differed only in the amount of TMPTA cross-linker present (monomer:TMPTA molar ratios of 10:1.0 vs 10:0.8, respectively): cross-linked **P8** was the most rigid, whereas cross-linked **P10** was the most deformable. The attempted cross-linking of organic polymer **P5** using the same TMPTA and initiator stoichiometries produced a material too fragile to form a film suitable for tensile testing.

Photochemical cross-linking was also used to stabilize materials processed by direct ink writing (DIW) 3D printing. The **P2** composite showed rheological behavior advantageous for this application, with the storage modulus (*G*′) exceeding the loss modulus (*G*″) at low strains, followed by a crossover point at higher strains, indicative of yielding behavior (Figure S12A). In addition, steady shear measurements revealed a clear decrease in viscosity with an increasing shear rate (Figure S12B), confirming pronounced shear-thinning behavior. A corresponding ink performed well in a standard DIW format (Figure 3) and was easily photocured with a hand-held 405 nm ultraviolet lamp.

**Figure 3:**
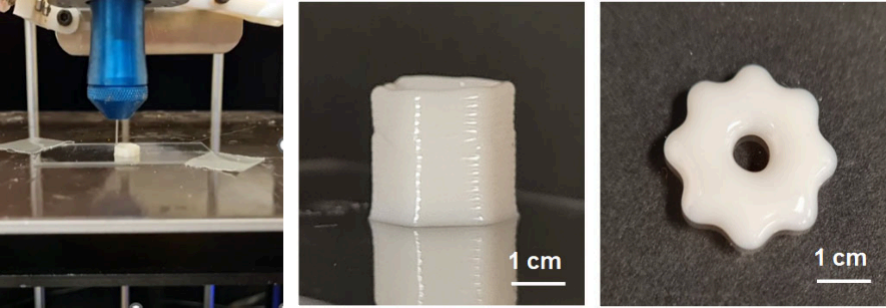
DIW 3D printing of **P2**/TMPTA (**P8** in Table 2) in cylindrical and flower shapes, shown after curing under UV–visible light.

Depolymerization and repolymerization: Reversible polymerization/depolymerization is characterized by the ceiling temperature (*T*_c_) at which the entropy and enthalpy balance to reach equilibrium. Since polymer **P5** has a reported *T*_c_ of 58 °C,^23^ we were able to depolymerize the composite materials under mild conditions, as shown in Figures 1 and S7 and summarized in Table 3. Composite **P2** was slow to decompose, generating only 5–10% of monomer in the absence of dodecanethiol in 1 h (entries 1–2). The addition of catalytic thiol greatly enhanced this process, resulting in near-complete depolymerization of **P2** and the recovery of nearly 70% of monomer **1** by column chromatography (Figure S8). This monomer was smoothly repolymerized to regenerate **P2** and **P8** with very similar NMR spectra (Figure S9) and physical properties (Figure S10). As reported by Gutekunst and colleagues, the linear polymer **P5** underwent depolymerization to a similar extent under the same thiol-catalyzed conditions.^23,39^

**Table 3:** Depolymerization Reactions

entry	sample	additives^a^	conversion^b^ (%)	isolated yield (% monomer)
1	**P2**		5–10	
2	**P2**	DBU	10 ± 5	
3	**P2**	DBU/H_25_C_12_-SH	87 ± 5	68 ± 5
4	**P8** ^c^	DBU/H_25_C_12_-SH	nd	40 ± 10

a N_2_ atmosphere; THF solvent; [polymer] = 25 mM in repeat unit; [DBU] = 0.25 mM, [dodecanethiol] = 0.25 mM when used; 62 °C; 1 h (12 h for entry 4).

b Determined by ^1^H NMR of the crude reaction mixture cooled to room temperature.

c Insoluble, so heated as a suspension.

Heating of a suspension of cross-linked material **P8** and the 3D-printed version of the same material netted 40 ± 10% of isolated **1** after 12 h at 62 °C. The reduced yield relative to **P2** can be attributed to the formation of thiol adducts with TMPTA, which require much higher temperatures to reverse. As expected, when used in polymerization reactions to remake **P2** and **P8** in three cycles of polymerization/depolymerization, the recovered monomer performed identically to the starting monomer. In contrast, direct repolymerization of the crude depolymerized liquid, initiated by the addition of DBU and additional TMPTA produced yellowed and more brittle cross-linked material that was not film-forming.

Ring-opening polymerization of a cyclic thiolactone has been implemented with thiol-functionalized silica particles (SiO_2_@SH) as macroinitiators and augmented by cross-linking via conjugate addition. Such covalent modifications require fast and strongly thermodynamically driven reactions to overcome entropic and surface-accessibility constraints on bond formation on the surfaces of nanoparticle additives. The combination of these added components provided a broad range of properties. Covalent cross-linking was also used to provide convenient 3D printability. Depolymerization of the silica-thiol-initiated materials gave recyclable monomer in high yields, whereas covalent cross-linking to acrylate cut such yields approximately in half.

While the effects were relatively modest in magnitude, the most important aspect of this work so far is the demonstration that covalent integration of a silica filler can produce measurable enhancements of bulk thermal and mechanical properties relative to the filler alone. We believe that two factors may be at play, both impacting the way in which the hard nanoparticles and soft bulk polymer matrix interact at their interface. The most obvious difference is the presence in **P2** and **P8** of covalent silica–polymer bonds, which must differ from noncovalent interactions in strength and dynamic behavior. In addition, surface modification of silica nanoparticles reduces particle surface polarity and surface energy,^40^ which can significantly change particle agglomeration or dispersion. These factors remain to be independently addressed and augmented in follow-on studies.

## Supplementary Material



## Notes

Experimental details of syntheses, 3D printing, depolymerization, measurement of thermal and mechanical properties, and characterization data (NMR, XPS, TGA, bulk mechanical properties) (PDF)
