# Supplementary material for: Modulation of Depolymerizable Poly(thioether-thioester) Properties in Reversible Covalent Composites
Source: ACS Macro Lett. 2025 Sep 19;14(10):1425–30. doi: 10.1021/acsmacrolett.5c00422 (PMC12548341; doi:10.1021/acsmacrolett.5c00422)
Supplement: Supplementary file 1 [file mz5c00422_si_001.pdf]

Modulation of Depolymerizable Poly(thioether-thioester) Properties in Reversible Covalent Composites

Binoy Maiti,[a] Mridula Nandi,[a] Jaehyun Cho,[b] Liang Yue,[c] Kellie Stellmach,[a] Blair Brettmann,[b] Qi Jerry,[C] Will Gutekunst,[a] M.G. Finn*[a]

[a]School of Chemistry and Biochemistry, [b]School of Materials Science and Engineering, [c]George W. Woodruff School of Mechanical Engineering,
Georgia Institute of Technology, Atlanta, GA 30332, USA.

*Corresponding author email: mgfinn@gatech.edu

Supporting Information

Materials and Methods

All reagents and solvents were purchased from commercial suppliers and used as received: 1,2-ethanedithiol; triethylamine; α-bromoisobutyryl bromide; methyl isobutyl ketone; (3-mercaptopropyl)trimethoxysilane (MPS); trimethylolpropane triacrylate (TMPTA); 2,2-dimethoxy-2-phenylacetophenone (DMPA); 1-dodecanethiol; 1,8-diazabicyclo[5.4.0]undec-7-ene (DBU). Spherical silica particles (20 nm) were purchased from Millipore Sigma.

Syntheses

Monomer 1. This dithiolactone was prepared according to a modified literature method.[1] A round-bottommed flask was equipped with a dropping funnel and charged with dry CH_2Cl_2 (300 mL), 1,2-ethanedithiol (10.1 mL, 120.0 mmol, 1.0 equiv), and triethylamine (33.5 mL, 240.0 mmol, 2.0 equiv), and the reaction mixture was placed under N_2 atmosphere. After cooling in an ice bath to 0 °C, a solution of α-bromoisobutyryl bromide (14.8 mL, 120.0 mmol, 1.0 equiv) in dry CH_2Cl_2 (50 mL) was added dropwise over 1 h. The mixture was stirred for another 48 h at room temperature, the resulting white precipitate was filtered, and 200 mL of ice-cold 1 M HCl was added to the filtrate. The organic phase was washed with brine (2 x 300 mL), dried over anhydrous Na_2SO_4, and the solvent removed by rotary evaporation. The desired compound was purified by vacuum distillation (90°C, 0.26 mm Hg), to afford **1** as a colorless liquid. NMR of this material showed an unknown contaminant as well as a small amount of ethanedithiol. Pure material was obtained by elution through a plug of silica gel, eluting with 4% EtOAc in hexanes.

SiO_2@SH. Thiol-modified SiO_2 was synthesized according to a modified literature method.[2] In a 250 mL dried round-bottommed flask, 2 g of silica nanoparticles was dispersed in 125 mL of methyl isobutyl ketone by sonication for 20 min. (3-Mercaptopropyl)trimethoxysilane (MPS, 1 g) was added and the mixture was sonicated for an additional 10 min. The mixture was then heated at 110 °C with stirring for 16 h under N_2 atmosphere, cooled, and half of the ketone solvent was removed by rotary evaporation. The resulting thiol-modified silica nanoparticles (SiO_2@SH) were purified by five cycles of centrifugation, pouring off of solvent, and suspension in 1:1 acetone:hexanes, followed by drying under high vacuum overnight.

SiO_2@SH – polymer composites. In typical example, SiO_2@SH particles (480 mg, 12 wt% with respect to monomer) were sonicated in 3 mL dry CH_2Cl_2 in a 50 mL round-bottommed flask. The solution was then cooled in an ice-water bath. To the cooled solution, thiolactone monomer (4.0 g, 24.7 mmol) in dry CH_2Cl_2 (1 mL) and DBU (37.0 mg, 0.24 mmol) in dry CH_2Cl_2 (1 mL) were added sequentially, and the resulting reaction was stirred for 5-12 h under N_2 atmosphere. After completion,

the reaction mixture was quenched with one drop of trifluoroacetic acid. The solvent was evaporated at room temperature and the resulting solid product was precipitated five times from cold MeOH to remove residual monomer.

Crosslinked polymer composites. In a typical example, compound **P1** (1.2 g, 7.4 mmol), trimethylolpropane triacrylate (TMPTA, 219 mg, 0.74 mmol) and Igacure 819 (31 mg, 0.074 mmol) were dissolved in CH_2Cl_2 (1.5 mL). The mixture was transferred to a PTFE block (4 cm x 2 cm x 0.5 cm) and the solvent was allowed to evaporate at room temperature. The resulting sample was then irradiated (365 nm) for 2-5 min to produce crosslinked film.

Samples of initial weight W_0 (excluding the weight of the Igacure initiator) were extracted with THF for 24 h to remove soluble species, and the resulting polymeric material was dried under vacuum at 60 °C until constant weight (W_{gel}) was reached. The gel fraction (%), calculated as (W_{gel}/W_0) × 100%, was found to be 72 ± 1% for two independent experiments, confirming the incorporation of thiol-functionalized silica particles into the acrylate network (or, conversely, crosslinking of thiol-functionalized silica particles by tripodal acrylate monomer or oligomers).

3D printing of polymer composite

Irgacure 819 (1 wt%) was added into the viscous polymer mixture as a photoinitiator. After thorough mixing, the ink was loaded into a 10 cc syringe and centrifuged at 4000 rpm for 10 min to remove air bubbles. The ink was then printed (10 mm/s) with a custom built DIW printing platform (Advanced Materials 34 (39), 2204890) using a 20-gauge nozzle (0.603 mm inner diameter) with a pressure of 15 psi. The printed structures were then photocured with a 405 nm UV lamp. The printing resolution depends on both the nozzle size and the precision of the moving stage. For the custom-built printing platform used in this work, the achievable resolution is approximately 50 µm.

Depolymerization of polymer composites

In an oven-dried 50 mL sealed tube, 1.0 g of composite was dissolved in dry THF. DBU and dodecanethiol (0.01 equiv each) were added and the mixture was cooled in an ice bath and purged with N_2 for 15 min. The mixture was heated at 62 °C for 1 h, monitoring by ^{1}H NMR and TLC. After completion of the reaction, the reaction mixture was cooled and purified by column chromatography (eluting with 4% EtOAc in hexanes) to recover the monomer in 68-70% isolated yield. The **P8** crosslinked membrane was subjected to the same depolymerization conditions, except heating was maintained for 12 h and the monomer was isolated in 40±10% yield.

Thermal Properties

Thermal stabilities and decomposition temperatures (T_d) were determined by thermogravimetric analysis (TGA) using a Mettler TGA 2 Star System. This instrument requires 3-5 mg of sample; in an alumina crucible and heated from 50 °C to 700 °C at a rate of 10 °C/min under N_2 flow.

Differential scanning calorimetry (DSC) was performed on a TA Instruments DSC250 calorimeter. For non-isothermal measurements, the samples were subjected to an initial heating cycle to eliminate their thermal history and residual solvent, followed by cooling and a second heating cycle from which the reported data was obtained. Initial cycle: -30 °C to 100 °C at a heating rate of 10 °C/min, held for 2 min at 100 °C, followed by cooling to -40 °C at 10 °C/min, held for 5 min at -40 °C; second cycle: heating at 10 °C/min. The reported glass transition temperature (T_g) is the midpoint of the step change in heat flow observed during the second heating cycle.

Mechanical Properties

Uniaxial tensile tests were performed using a universal testing machine (UTM, Insight 10, MTS Systems Corp., Eden Prairie, MN, USA) with a cross-head speed of 10 mm/min. Sample dimensions

= 5 mm (width) x 25 mm (length) x 1 mm (thickness). Measurements were done at least five times for each sample, results being averaged and the corresponding standard deviations considered.

Rheological characterization (Fig. S11) was performed using a rotational rheometer (Discovery HR-2, TA Instruments, New Castle, DE, USA) at 25 °C. A 40-mm-diameter cross-hatched plate with a 0.7 mm gap height was employed for testing. Steady-state flow experiments determined apparent viscosity across shear rates ranging from 0.1 to 400 s^{-1}. Oscillation experiments evaluated shear storage modulus (G′) as a function of strain (0.02%–5000%) at a fixed frequency of 1 Hz.

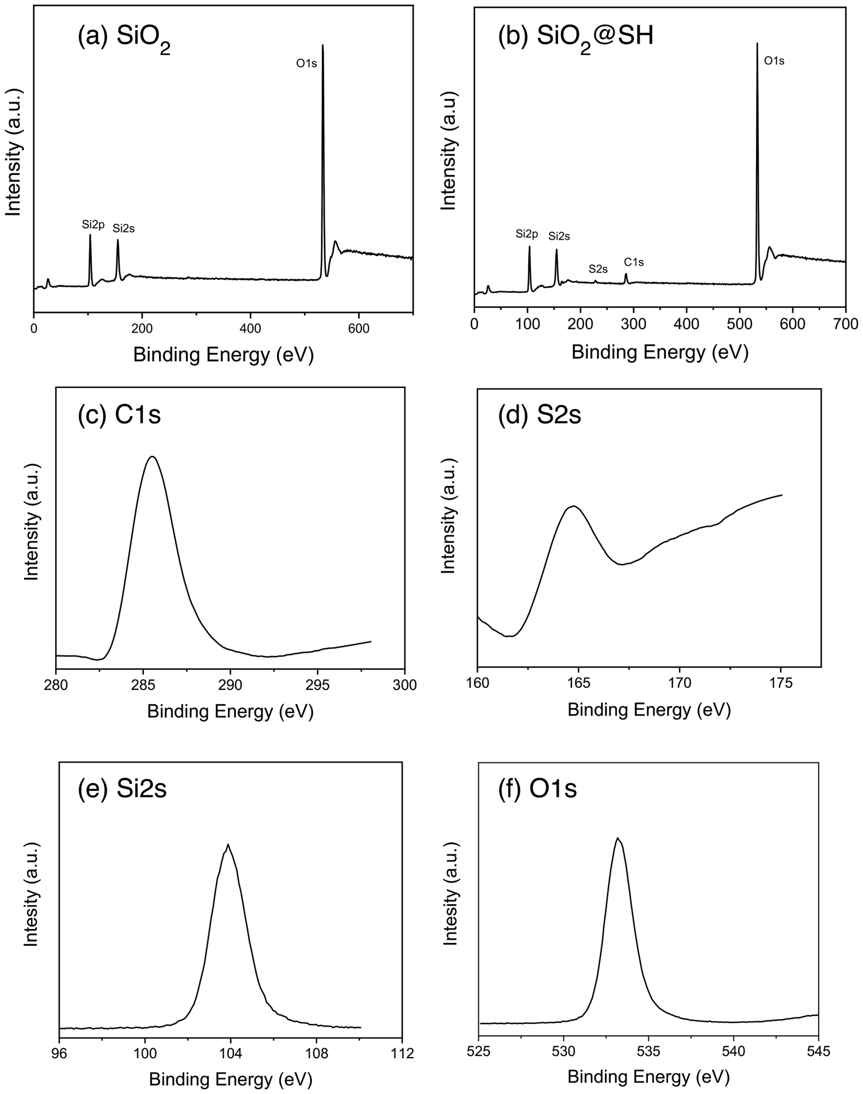

Figure S1. XPS (a) wide scan of the bare SiO_2, (b) wide scan of SiO_2@SH, (c-f) C1s, S2s, Si2s, and O1s core-level peaks of SiO_2@SH.

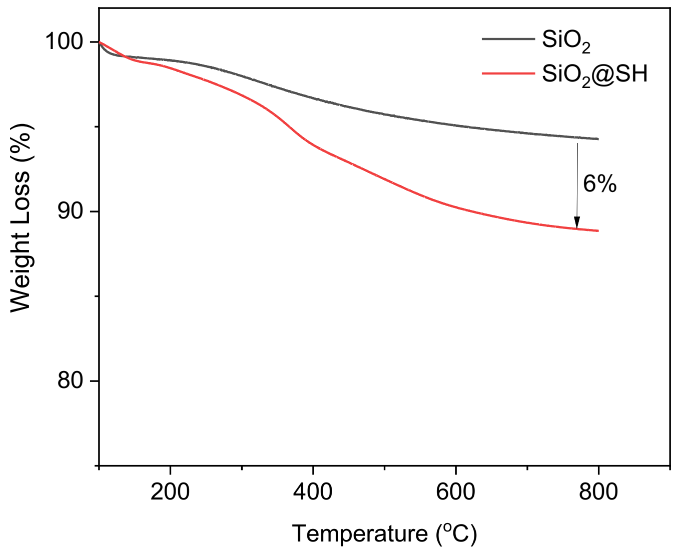

Figure S2. TGA curves of SiO_2 and SiO_2@SH.

The amount of MPS present on the SiO_2 particle was calculated from the above data (6% weight loss) using the following equation 1.[3, 4]

$$\text{Attached amount (mmol/g)} = \frac{1000\Delta W}{(100-\Delta W)M} \qquad \text{(eqn. 1)}$$

Where M (g/mol) is the molar mass of the degradable part (MPS) of the grafted molecule. $\Delta W = [W_{\text{silica(200-700)}} - W_{\text{Thiol gr. 200-700)}}]$

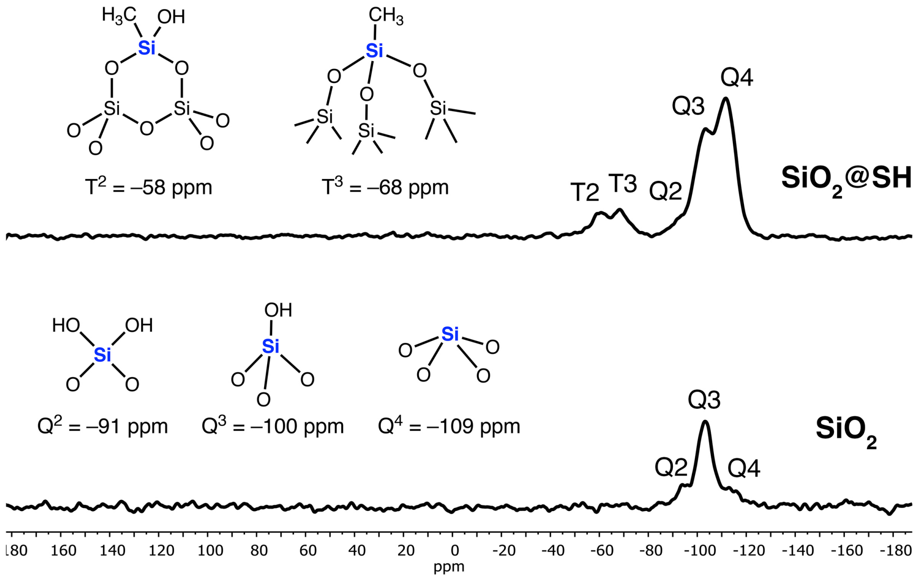

Figure S3. ^{29}Si MAS NMR spectra of SiO_2 and SiO_2@SH.

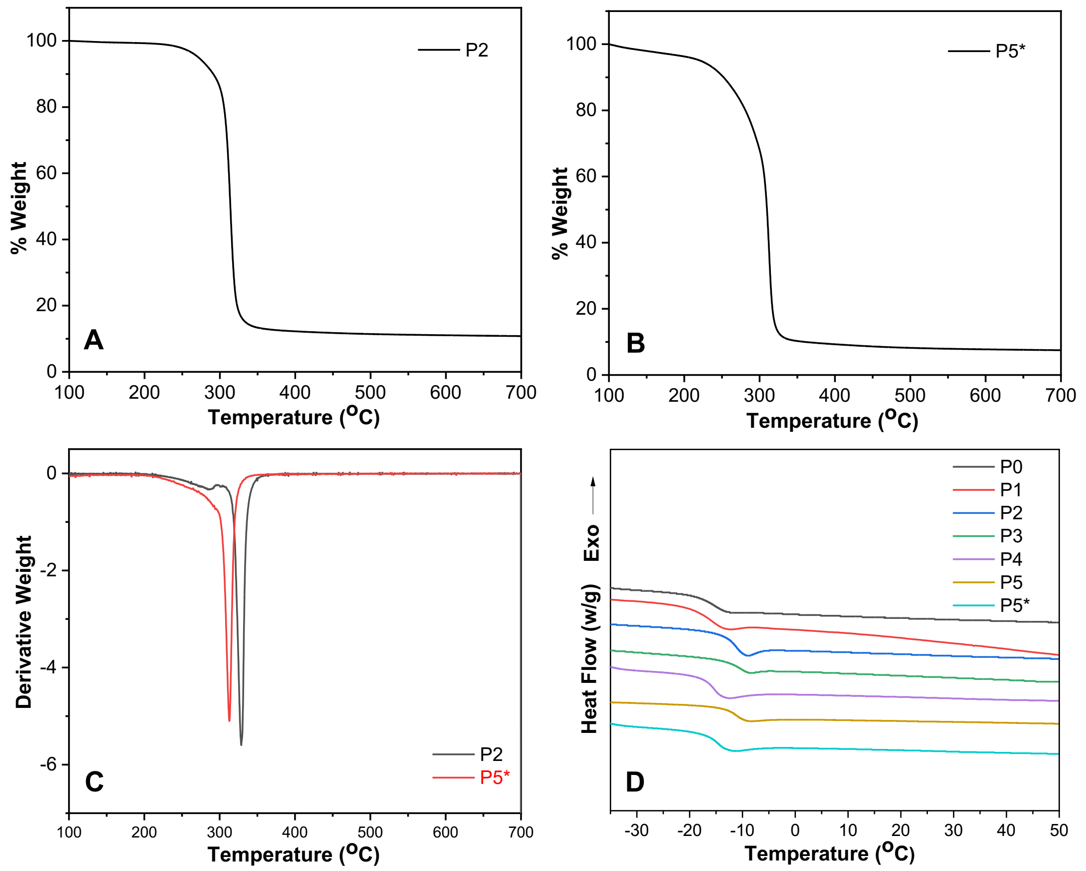

Figure S4. Representative TGA thermograms of **P2** (A) and **P5***(B), (C) DTG of **P2** and **P5*** and (D) DSC of the indicated materials. TGA thermograms of the replicates and other composites are provided in the supplementary information.

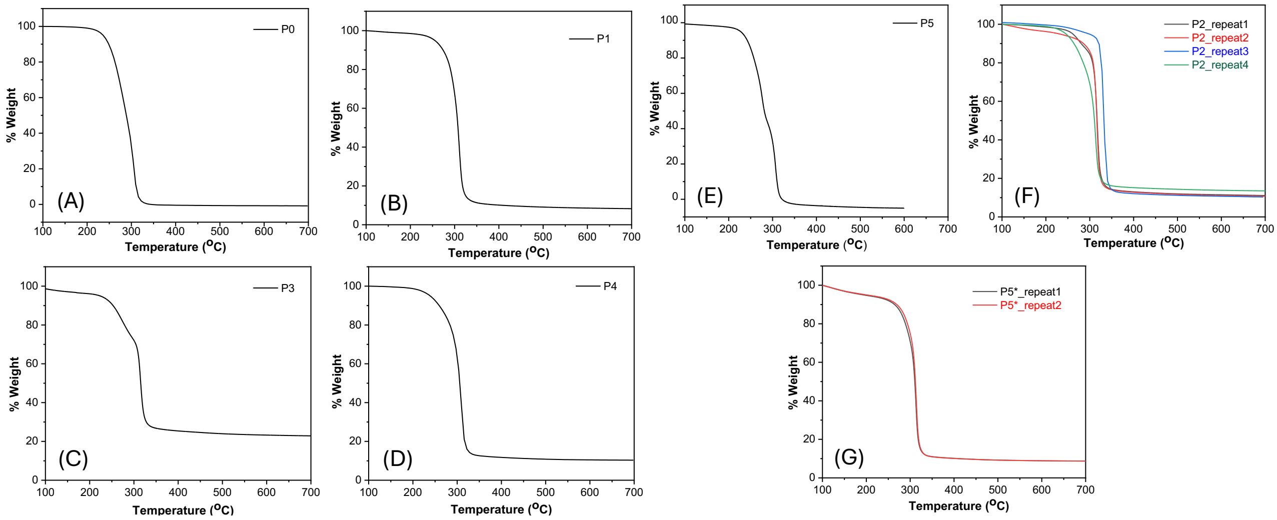

Figure S5. Representative TGA analyses of (A) **P0**, (B) **P1**, (C) **P3**, (D) **P4**, (E) **P5**, and replicates of (F) **P2** and (G) **P5***.

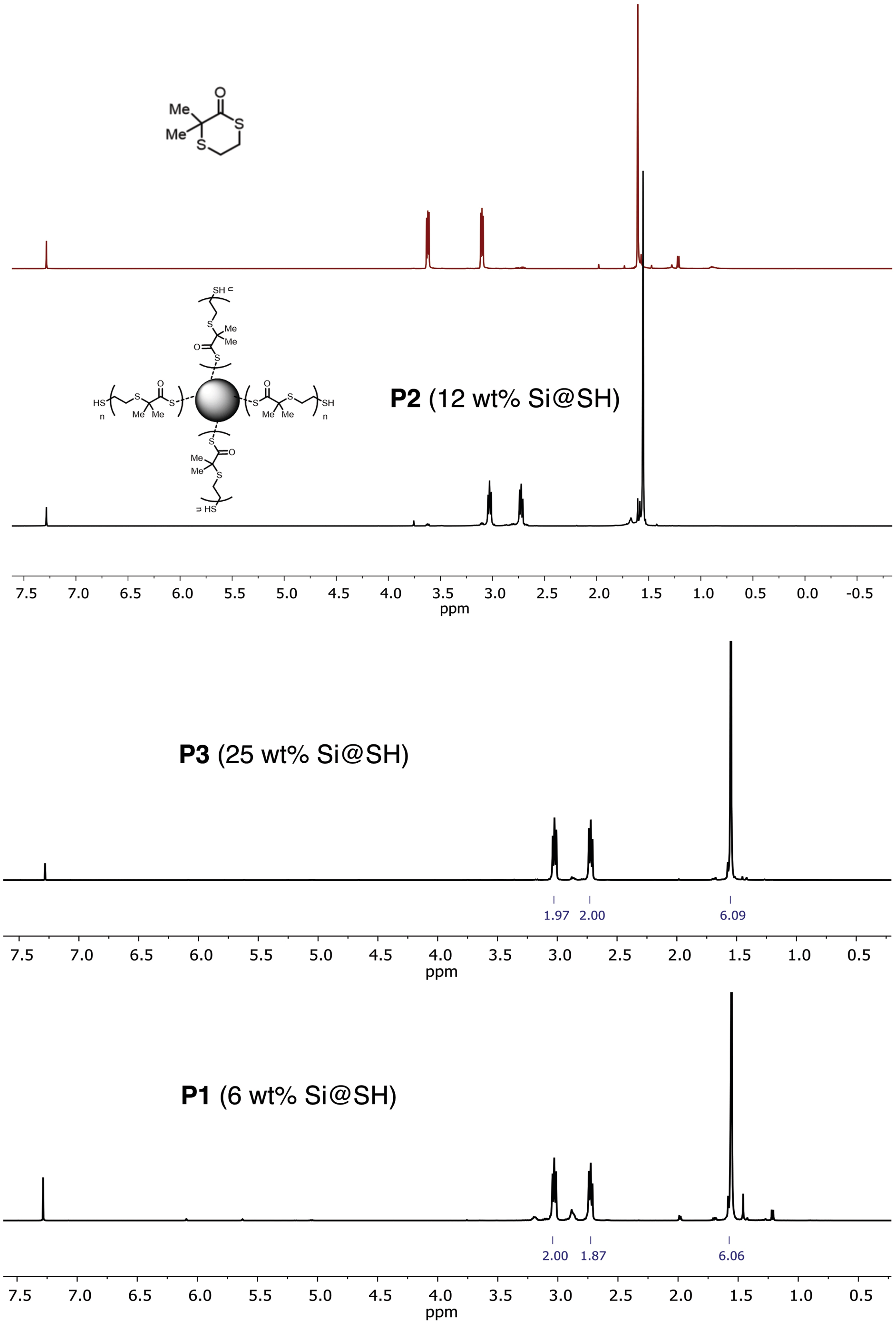

Figure S6. ^{1}H NMR of dithiolactone **1** and SiO_2@SH polymer composites in $CDCl_3$.

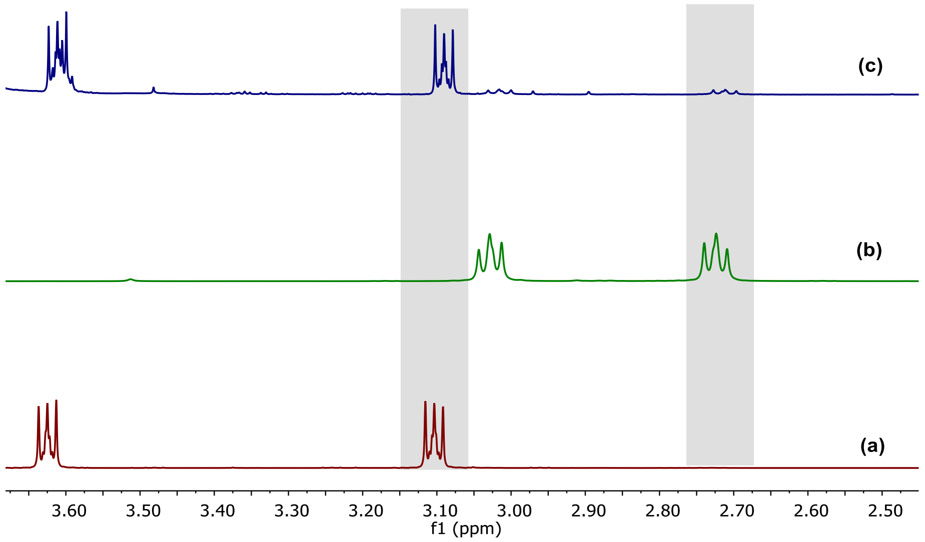

Figure S7. ^{1}H NMR spectra of (a) monomer **1**, (b) **P2** (Table 3, entry 3) and (c) after 1 h of heating a solution of **P2,** [polymer] = 25 mM in repeat unit in the presence of DBU (0.25 mM) and dodecanethiol (0.25 mM).

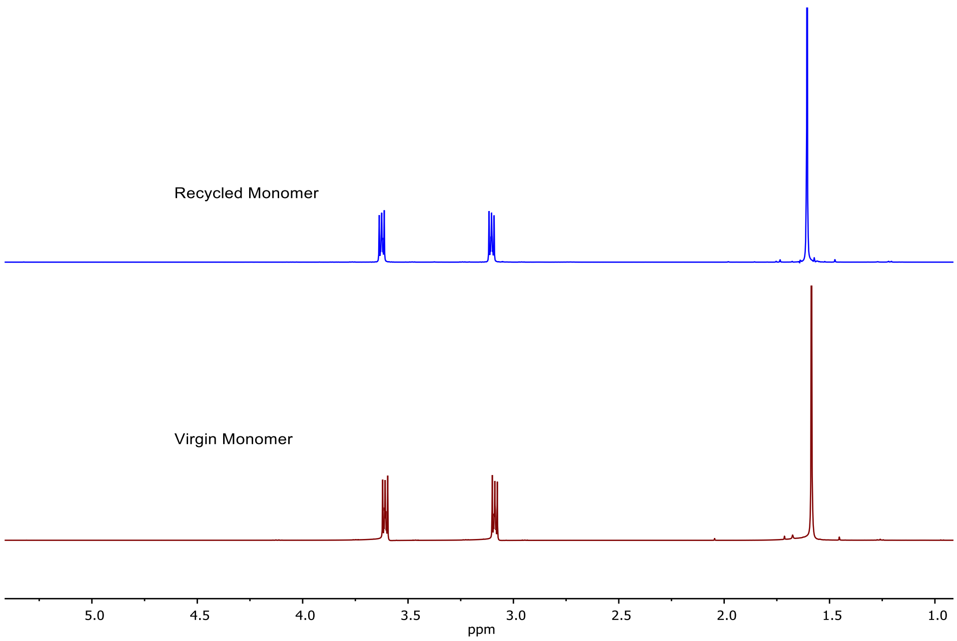

Figure S8. Overlay of ^{1}H NMR spectra in $CDCl_3$ of started dithiolactone (bottom) and recycled dithiolactone after depolymerization (top).

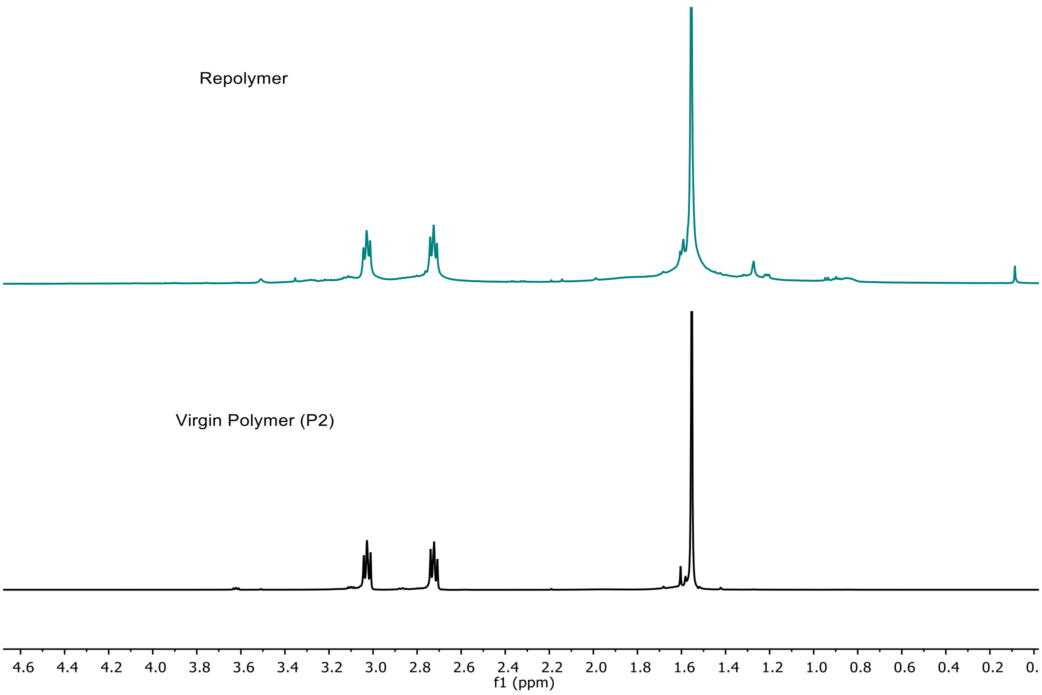

Figure S9. Overlay of the ^{1}H NMR spectra in $CDCl_3$ of **P2** (bottom) and the same material re-made from recovered after depolymerization monomer (top).

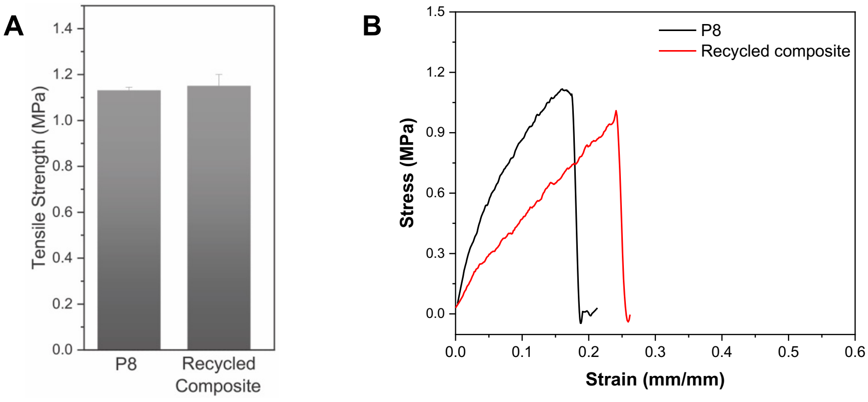

Figure S10. Tensile strength (A) and stress-strain to break (B) of virgin **P8** and the same material prepared from recovered monomer.

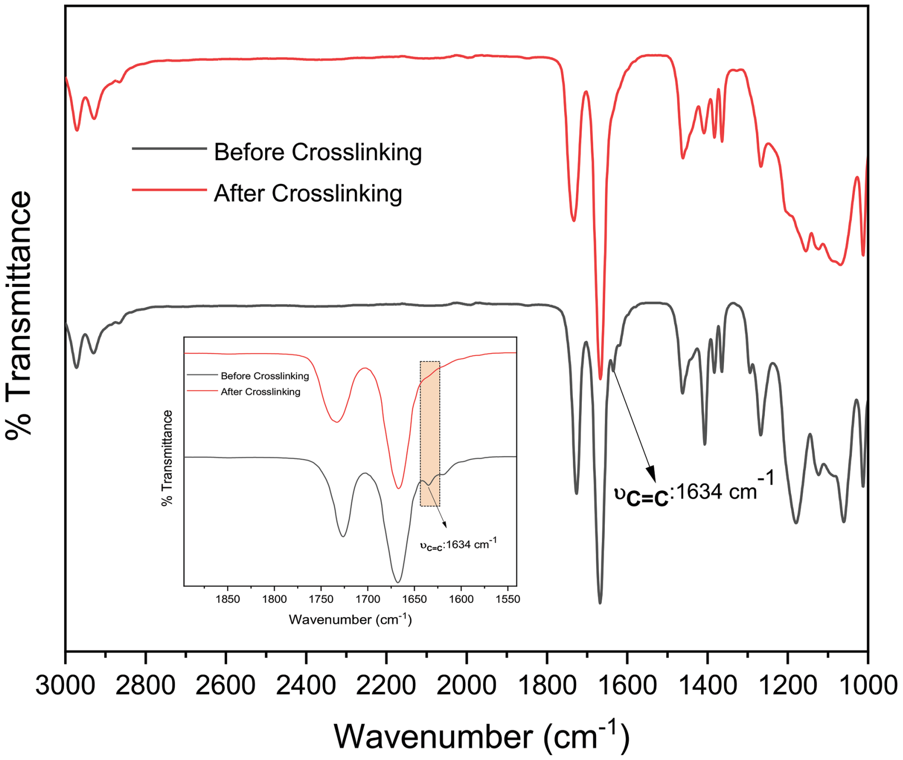

Figure S11. FTIR spectra of the composite mixture before and after crosslinking under UV irradiation to make **P8**.

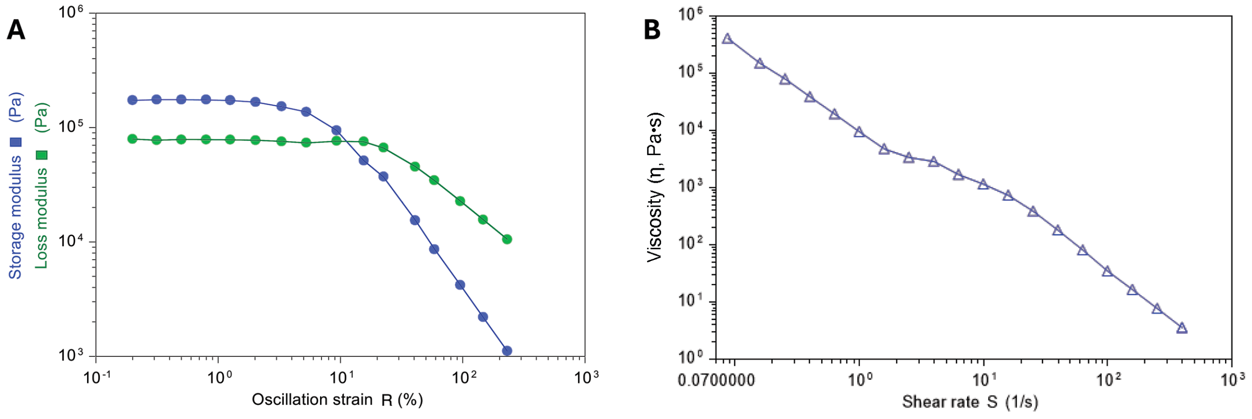

Figure S12. Rheological characterization of the **P8** polymer composite. (A) Shear storage and loss moduli of the ink. (B) Apparent viscosity of the ink, showing shear thinning.

Table S1. Summary of thermal properties of replicates of polymer composites (P2 and P5*) from TGA analyses.

Sample Name	Composition	T_{onset} (°C)	T_{D50} (°C)	T_{max} (°C)
**P2**	**1**/ SiO_2@SH (12 wt%)	303	314	329
**P2_Repeat1**	**1**/ SiO_2@SH (12 wt%)	307	317	318
**P2_Repeat2**	**1**/ SiO_2@SH (12 wt%)	308	316	316
**P2_Repeat3**	**1**/ SiO_2@SH (12 wt%)	299	311	328
**P5***	**P5**/ SiO_2 (12 wt%)	301	310	313
**P5*_Repeat1**	**P5**/ SiO_2 (12 wt%)	302	310	314
**P5*_Repeat2**	**P5**/ SiO_2 (12 wt%)	304	312	315

References

(1) Stellmach, K. A.; Paul, M. K.; Xu, M.; Su, Y.-L.; Fu, L.; Toland, A. R.; Tran, H.; Chen, L.; Ramprasad, R.; Gutekunst, W. R. Modulating Polymerization Thermodynamics of Thiolactones Through Substituent and Heteroatom Incorporation. *ACS Macro Letters* **2022**, *11*, 895-901. doi 10.1021/acsmacrolett.2c00319.

(2) Han, Y.; Liu, M.; Li, X.; Liang, P.; Song, Y.; Qiao, X. Polyhedral oligomeric silsesquioxane grafted silica-based core-shell microspheres for reversed-phase high-performance liquid chromatography. *Microchimica Acta* **2019**, *186*, 331. doi 10.1007/s00604-019-3441-6.

(3) Zabihi, O.; Khayyam, H.; Fox, B. L.; Naebe, M., Enhanced thermal stability and lifetime of epoxy nanocomposites using covalently functionalized clay: experimental and modelling. *New J. Chem.* **2015,** *39*, 2269-2278.

(4) Zabihi, O.; Ahmadi, M.; Abdollahi, T.; Nikafshar, S.; Naebe, M., Collision-induced activation: Towards industrially scalable approach to graphite nanoplatelets functionalization for superior polymer nanocomposites. *Sci. Rep.* **2017,** *7*, 3560.
